# Serology in the Digital Age: Using Long Synthetic Peptides Created from Nucleic Acid Sequences as Antigens in Microarrays

**DOI:** 10.3390/microarrays5030022

**Published:** 2016-08-10

**Authors:** Muhammad Rizwan, Bengt Rönnberg, Maksims Cistjakovs, Åke Lundkvist, Rudiger Pipkorn, Jonas Blomberg

**Affiliations:** 1 Section of Clinical Virology, Department of Medical Sciences, Uppsala University, Uppsala 751 85, Sweden; rizwan.muhd715@gmail.com (M.R.); bengt.ronnberg@gmail.com (B.R.); Maksims.Cistjakovs@rsu.lv (M.C.); 2 Laboratory of Clinical Microbiology, Uppsala University Hospital, Uppsala 751 85, Sweden; ake.lundkvist@imbim.uu.se; 3 August Kirchenstein Institute of Microbiology and Virology, Riga Stradins University, Riga LV-1067, Latvia; 4 Zoonosis Science Center, Department of Medical Biochemistry and Microbiology, Uppsala University, Uppsala 75123, Sweden; 5 Deutsches Krebsforschungszentrum, Heidelberg 69121, Germany; pipkorn@t-online.de; 6 Laboratory of Clinical Microbiology, Linköping University Hospital, Linköping 58185, Sweden

**Keywords:** megapeptide, suspension microarray, immunoassay, suspension multiplex immunoassay (SMIA), emerging virus infections

## Abstract

Background: Antibodies to microbes, or to autoantigens, are important markers of disease. Antibody detection (serology) can reveal both past and recent infections. There is a great need for development of rational ways of detecting and quantifying antibodies, both for humans and animals. Traditionally, serology using synthetic antigens covers linear epitopes using up to 30 amino acid peptides. Methods: We here report that peptides of 100 amino acids or longer (“megapeptides”), designed and synthesized for optimal serological performance, can successfully be used as detection antigens in a suspension multiplex immunoassay (SMIA). Megapeptides can quickly be created just from pathogen sequences. A combination of rational sequencing and bioinformatic routines for definition of diagnostically-relevant antigens can, thus, rapidly yield efficient serological diagnostic tools for an emerging infectious pathogen. Results: We designed megapeptides using bioinformatics and viral genome sequences. These long peptides were tested as antigens for the presence of antibodies in human serum to the filo-, herpes-, and polyoma virus families in a multiplex microarray system. All of these virus families contain recently discovered or emerging infectious viruses. Conclusion: Long synthetic peptides can be useful as serological diagnostic antigens, serving as biomarkers, in suspension microarrays.

## 1. Introduction

The frequent appearance of novel pathogens [1,2,3,4,5], and reemergence of those who became rare [6], emphasizes the need for an ability to generically detect them, and rapidly evolve diagnostic techniques that can enhance the response to such challenges. Antibody detection (serology) provides a unique possibility to detect past events encountered by the immune system, like an infection or exposure to an antigen mimicking an autoantigen. Nucleic acid detection of a microbe cannot do this, unless the microbe causes an ongoing infection. Moreover, the amplification inherent in the immune response means that antibody detection can be more sensitive than nucleic acid detection. The infecting pathogen is often sequestered in the body and not accessible for sampling, but the antibodies to it are available in blood and can often be readily detected many years after the infection. Thus, nucleic acid and antibody detection go hand in hand for diagnosis and surveillance of many medically important infectious diseases. Antibodies to a pathogen are traditionally detected to one antigen at a time using agent-specific ELISA (enzyme-linked immunosorbent assays). However, many different antibodies can now be detected simultaneously, e.g., with SMIA (suspension multiplex immunoassay) [7,8,9,10], a rational and economic technique. SMIA is a cost-effective serological method where antigen and serum consumption can be minimized, its background is generally low, and the dynamic range wider than in ELISA. Unlike the latter, it provides multiple results per analysis [7,8,9,10].

There are a significant number of severe infections that can be difficult to diagnose without appropriate serological information, which calls for diagnostic arrays covering many differential diagnoses. The increased travel between continents increases the need for geographically-unrestricted diagnostic panels.

Moreover, the frequent appearance of novel pathogens emphasizes the need for an ability to generically detect, and rapidly evolve, diagnostic techniques that can efficiently respond to such challenges. Antigens for such serological diagnostic systems optimally need to be prepared without any delay. Novel pathogens are now often identified through rapid sequencing [5,11,12]. It is then a non-trivial bioinformatic task to identify suitable antigens from the sequence. Such antigens can be produced as recombinant proteins from synthetic nucleic acid, or as synthetic peptides. The ability to re-create the original antigenic structure of the microbial sequence, including both linear and conformational epitopes, generally increases the greater the portion of the antigen that is part of the synthetic construct, in essence, converting linear (one-dimensional) information to three-dimensional. This is the rationale behind the “megapeptide” concept described here.

In the following, we give examples of the bioinformatic selection and experimental evaluations of diagnostically-useful large synthetic antigens. The diagnostic antigens were obtained from sequencing of targeted pathogens and they were produced in a form suitable for SMIA.

## 2. Materials and Methods

### 2.1. Megapeptides

The peptide sequences are detailed in Table S1. All had the spacer NH_2_-PEG_6_-His_6_-PEG_6_ at their amino terminus, where PEG_6_ is hexaethylene glycol and His_6_ is hexahistidine (enabling coupling control via anti-His antibodies). The peptides were synthesized under optimized conditions for long peptides (Pipkorn, unpublished). Briefly, solid phase synthesis was done on an automated multiple-peptide synthesizer (AMS 422; Abimed Analysen-Technik, Langenfeld, Germany), using coupling times and conditions optimized for high coupling yield. For amino-terminal protection, the base-labile FluorenylMethylOxyCarbonyl chloride (FMOC) group was used. In a few cases, megapeptides were subsequently analyzed by reverse-phase high-performance liquid chromatography (HPLC) on a C18 column. They yielded several peaks, indicating non-homogeneity (unpublished data). Lyophilized peptides were dissolved in dimethyl sulfoxide (DMSO) (Sigma-Aldrich Inc., Saint Louis, MO, USA, #Sigma-D2650).

The epitope prediction tools at the Immune Epitope Database [13] and 3D structures found in the Protein Data Bank [14] were utilized for optimization of megapeptide design.

### 2.2. Other Reagents

In the Ebola study, control antigens were recombinant glycosylated E proteins of the Sudan (SEBOV), Zaire Ebola (ZEBOV), Reston, and Bundibugyo strains of Ebola virus, and Marburg virus. Control sera were rabbit anti-Bundibugyo, anti-SEBOV, and anti-ZEBOV glycoproteins, respectively. They were all purchased from Integrated BioTherapeutics Bioservices, Gaithersburg, MD, USA. A convalescent serum from a patient diagnosed with an uncertain filovirus infection was obtained with informed consent. Anonymous blood donor sera from the Uppsala Academic Hospital were used as negative controls. They were all used with informed consent according to the Swedish Biobank law which allows diagnostic patient samples to be used for similar purposes as the original sampling purpose.

In the HHV7 and JCV studies, we used 82 and 131 blood donor sera (respectively, see above) which contained HHV7 and JCV antibody reactivity.

### 2.3. Coupling of Peptides to Magnetic Luminex Beads

Luminex carboxylated differentially-color-marked magnetic microspheres (MagPlex-C microspheres, Luminex Corp., Austin, TX, USA) were used for the coupling of antigens. Coupling was performed according to the protocol provided by the manufacturer. See the sample protocol for two-step carbodiimide coupling of protein to magnetic beads [15]. A detailed account of the coupling procedure is out of scope for this paper. Briefly, the coupling was made with 200 µL of the stock microsphere solution containing approximately 1.25 × 10^7^ beads per ml. We have found the optimal quantity of antigen for coupling of 200 μL of Magplex microsphere suspension to be 5–10 μg of recombinant protein antigen and 50 μg of synthetic peptide antigens. However, it is possible to save reagents by using smaller amounts and volumes. All volumes in the recipe should then be decreased proportionally. After the coupling of antigen with beads, beads were incubated with 0.5 mL of PBS containing 0.05% (*v*/*v*) Tween 20 and 50 mM Tris (PBST) in the dark for 15 min on a rocking mixer at room temperature. The purpose of which was to block unreacted carboxyl groups with primary amines. The beads were then washed once with 0.5 mL StabilGuard (SurModics, Eden Prairie, MN, USA, #SG01-1000) using a magnetic separator. The bead pellet was finally resuspended in 400 µL StabilGuard. This created a bead mixture consisting of 6250 beads/µL. The coupled beads were stored at 4 °C in the dark. We found that coupled beads could be stored for at least a month (unpublished data). Prolonged storage at 4 °C can create problems due to antigen degradation, bacterial, or fungal growth. The beads with different numbers can be combined to make a serological panel for use in a multiplex system which theoretically could have a maximal multiplexity of 200 at a time. However, this becomes expensive due to antigen and bead costs. In our work, we have stayed at multiplexities below 50.

### 2.4. Suspension Multiplex Immunoassay (SMIA)

**Figure 1:**
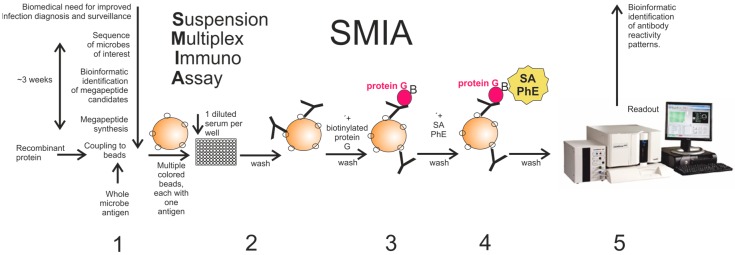
Flowchart of the events from the genome sequence of microbe until the final readout in the Luminex system and presentation of results. It shows a rough estimation of the time span from microbial genome sequencing and its processing for designing the megapeptides ready for use in SMIA. Antigens used here were whole purified microbe (virus), recombinant (glycosylated or non-glycosylated) protein, and synthetic peptide (30–140 amino acids; aa). SMIA steps are (**1**) covalent coupling of antigen to colored magnetic bead; (**2**) addition of antigen-coupled beads (the SMIA panel) to a diluted serum in a microplate well then incubated; (**3**) incubation with biotinylated-protein G; (**4**) incubation with SA-PhE; (**5**) readout of the microplate in the Luminex flow meter. The subsequent step, bioinformatic interpretation of the serological reactivity patterns, is not discussed in this paper. SA-PhE: Streptavidin-Phycoerythrin.

Fifty microliters of serum diluted 1/20 in phosphate buffered saline (0.15 M Sodium Chloride, 10 mM Sodium Phosphate; PBS), pH 7.4, containing 0.05% (*v*/*v*) Tween 20, 50 mM Tris and 2% (*v*/*v*) Prionex (Sigma-Aldrich Sweden AB, Stockholm, Sweden, #Sigma-Aldrich-81662) (PBSTP) was added to wells of a round bottom 96-well microtiter plate (Greiner Bio-One GmbH, Frickenhausen, Germany, #Greiner-104650) excluding the blank and controls. Fifty microliters of a vortexed and sonicated bead mixture consisting of 25 beads/µL suspended in PBSTP was then added to each well. The plate was then incubated in the dark with gentle rotation for 1 h at 37 °C. Post incubation, the wells were washed with PBS using a magnetic plate separator (Novex by Life technologies, Carlsbad, CA, USA #lifetechnologies-A14179). After that, the beads were resuspended in 50 µL of PBSTP and 50 µL of biotinylated-protein G (Pierce Biotechnology, Rockford, IL, USA, #Pierce-29988) at the concentration of (4 µg/mL of PBSTP) in each well. The plate was then placed for second incubation for 30 min at 37 °C in the dark with rotation. The wells were washed again with PBS and the beads were resuspended in 50 µL of PBSTP followed by the addition of 50 µL of streptavidin-phycoerythrin (SA-PhE) (Invitrogen-Thermo-Fisher, Waltham, MA, USA, #invitogen-S866) at the concentration of (4 µg/mL in PBSTP) in each well. The plate was placed for the final incubation for 15 min at 37 °C in the dark with rotation. The beads were washed once with PBS before they were resuspended in 100 µL of PBS and analyzed in a Luminex-200 (Luminex Corporation, Austin, TX, USA) instrument according to the instruction from the manufacturer. A minimum of 100 events for each bead number was set to read and the median value was obtained for the analysis of the data. All samples were analyzed in duplicate and average readings calculated. To detect any antibody binding to the beads themselves, a naked non-peptide-containing (“blank”) bead was included. A control His_6_ bead was also included in the SMIA reaction. The His_6_ tag allowed monitoring of coupling of the megapeptides to the magnetic beads using anti-hexahistidine antibodies (Antibodies- online, Atlanta, GA, USA, #ABIN100493). We did not observe false positive reactions due to anti-His_6_ in the sera tested for this paper. One negative control, where PBSTP instead of serum was added, was also used in all experiments. The work flow is presented in Figure 1.

## 3. Results

An overview of the SMIA serological serosurveillance and diagnostic tool is given in Figure 1.

### 3.1. Mimicking Ebola Glycoprotein Antigenicity with A Megapeptide

**Figure 2:**
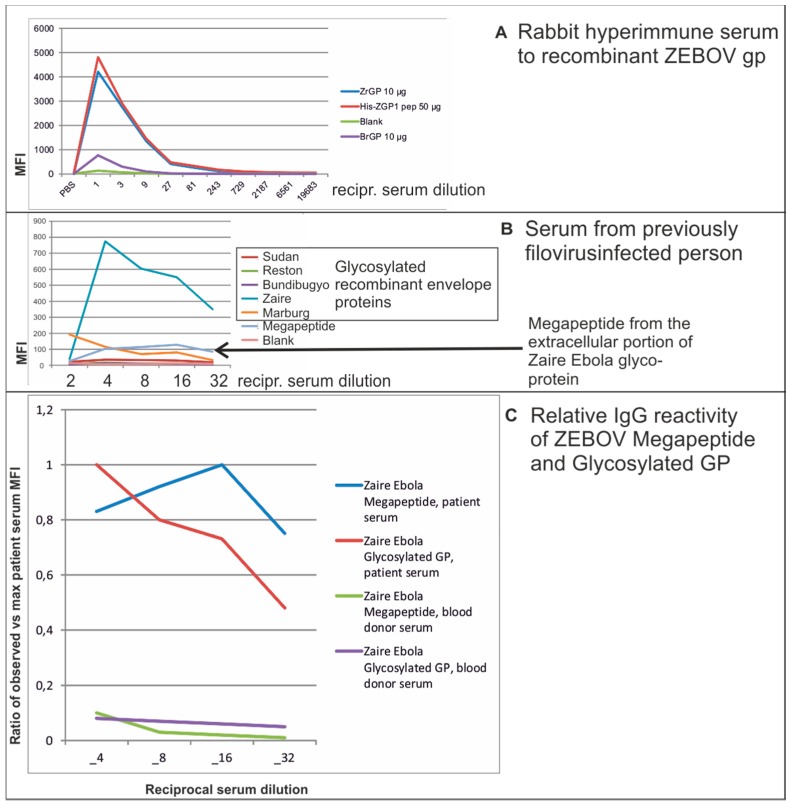
SMIA results with a megapeptide from the extracellular portion of the Zaire strain of Ebola virus (“megapeptide”, or “His-ZGP1 pep”), recombinant glycosylated glycoprotein from the Zaire and Bundibugyo strains of Ebola (“ZrGP” and “BrGP”, respectively). The megapeptide was coupled at a concentration of 50 µg/reaction while recombinant proteins were coupled at 10 µg/reaction. Results with dilutions of (**A**): a rabbit hyperimmune serum to ZrGP; and (**B**): a serum from a patient previously infected with a filovirus, are shown. *Y* axes: median fluorescence intensity (MFI). *X* axes: reciprocal serum dilution. A likely prozone effect was seen at the ½ dilution; (**C**): relative reactivity (observed vs maximum MFI) for reciprocal dilutions 4–32 of the same patient serum as shown in (**B**), and for a blood donor serum. All dilutions refer to the 50 µL of diluted serum which together with the 50 µL bead panel had a final volume of 100 µL. Thus, final dilutions were two times higher than the ones mentioned in the figure.

We evaluated if a 116 amino acid (aa) megapeptide derived from the extracellular portion of the surface glycoprotein of Zaire Ebola virus would function as an antigen in SMIA. We compared the reactivity with positive control, patient, and negative control serum, of the megapeptide and a glycosylated recombinant protein from the same viral strain and from related filoviruses (Figure 2).

All 40 negative control sera (Swedish blood donors) were negative in the test (unpublished data). The positive rabbit control serum reacted as strongly with the megapeptide as with a recombinant glycoprotein from the same virus (Figure 2A). Dilutions of a serum from a person who had been infected with an unknown filovirus were analyzed against a panel of recombinant filovirus glycoproteins. Virtually all anti-glycoprotein IgG reactivity was to the recombinant Zaire Ebola glycoprotein (Figure 2B). A pronounced prozone effect was noted at the highest serum concentration. Such prozone effects can be caused by competition between high and low affinity antibodies at low serum dilutions. The low affinity antibodies are washed off in subsequent steps. In this case, the megapeptide reacted weaker than the glycosylated recombinant protein. However, the megapeptide reactivities were highly correlated with the recombinant protein reactivities, and reacted only weakly with Swedish blood donor sera (exemplified in Figure 2C). Thus, we demonstrated that a 116 aa Ebola megapeptide is potentially useful as one antigen in an Ebola serological panel.

### 3.2. Mimicking Human Herpesvirus 7 Glycoprotein B Antigenicity Using Megapeptides

**Figure 3:**
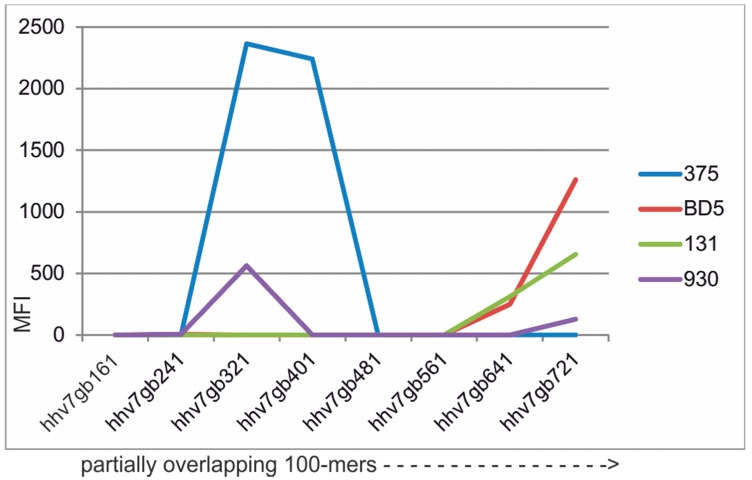
Distribution of IgG reactivity of four human sera with high HHV7 antibody activity, against overlapping 100-mers or longer (shown by dotted line with arrow) partially covering HHV7 glycoprotein B. Serum dilution 1/20 (cf. legend to Figure 2). Megapeptide names are explained in Table S1. Results from 82 sera are given in Table S2.

Human herpesvirus 7 (HHV7) is ubiquitous and is the least known of the nine human herpesviruses. It has mostly been associated with mild disease [16]. However, the diagnostic tools for its detection are not optimal and sensitive, and specific serology for detection of past and present HHV7 infection is needed. Glycoprotein B (gB) is a major antigenic herpesvirus protein. It is one of the most cross-reactive antigens among the herpesviruses. Cross-reactions occur primarily within the same herpesviral subfamily. In the HHV7 case, major sequence similarities and cross-reactions are with HHV6A, HHV6B, and CMV (*Betaherpesvirinae*). We strive to optimize herpesvirus serology and the mapping of optimal gB antigens for HHV7 is part of this. Figure 3 illustrates the result of a megapeptide scanning of most of HHV7 gB, using 82 sera. The results revealed that peptides covering the 300–450 and 600–721 amino acid regions were the most antigenic. Reactions were still relatively infrequent, though. In a putative “final” HHV7 serological panel, these HHV7 megapeptides must be complemented with other HHV7 antigens. Results with sera with high antibody reactivities to HHV7 are shown in Figure 3. These rather strong reactions contrasted with the minor and rare reactions of 30 aa peptides from several HHV7 proteins, including gB, in our tests (Table S2). The sum of the 7 reactivities above an arbitrary cutoff of 300 MFI for the 15 30mers was 4874 MFI (338 per peptide). The sum of the 18 reactivities above 300 MFI for the 8 megapeptides was 12949 MFI (1619 per peptide). Megapeptides thus gave more and stronger reactivities than the 30mers. The relation between the reactions of the megapeptides with those of other HHV7 antigens and other members of *Betaherpesvirinae* remains to be established.

### 3.3. Mimicking JC Polyoma Virus (JCV) Capsid Protein VP1 Antigenicity Using Megapeptides

**Figure 4:**
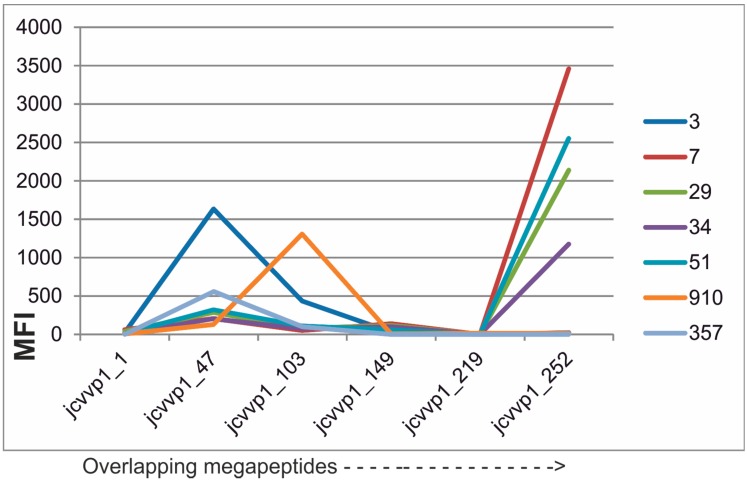
Distribution of IgG reactivity of seven human sera with high JCV antibody activity, against overlapping JCV VP1 102–116 aa megapeptides. Serum dilution 1/20 (cf. legend to Figure 2). Megapeptide names are explained in Table S1. Results with 130 sera are given in Table S3.

Another group of cross-reactive viral antigens are the capsid proteins (VP1) of human polyomaviruses [17]. Many new polyomaviruses have been discovered recently and there are currently 13 human polyomaviruses described. Most seem to be apathogenic for humans. However, some are associated with a panorama of pathologies ranging from encephalitis to cancer. The study of their epidemiology and pathogenesis can only be performed by reliable serological tools. JCV infections in immunosuppressed patients can be particularly serious. Sometimes, the deadly disease progressive multifocal encephalopathy is caused by JCV. It is imperative to create specific serological tools for this and for other important polyomaviruses. The capsid proteins are the major polyomaviral antigens. We therefore synthesized a set of six overlapping VP1 megapeptides from JC virus (Table S1). By SMIA, it turned out that the majority of IgG reactions were found against three megapeptides, amino acids 47–148, 103–218 and 252–354 (Figure 4), where reactivities of seven JCV antibody-positive blood donors are shown. Of the 131 sera, 32 (25%) did not react with an MFI of over 300 against at least one of the megapeptides (Table S3). These megapeptides are then antigen candidates for an HHV7 serological panel. Like among the herpesviruses, there are more or less pronounced cross-reactions between the capsid proteins of the polyoma viruses [17]. It is, therefore, reasonable to analyze polyoma antibodies in a “pan-polyomavirus” context. Whether a reaction is specific or nonspecific, often a cross-reaction, can then be sorted out using the most specific antigen(s) and the pattern of reactivity. The knowledge of epitope distribution presented here can now be used to create an optimal panel of synthetic polyomavirus antigens.

## 4. Discussion

### 4.1. Serology in the Digital Age

We developed new rapid and rational ways to produce antigens for microarrays, both what we call “megapeptides”, extraordinarily long synthetic peptides, in essence synthetic proteins (described in this paper), and by simple production of recombinant proteins [10].

By using megapeptides we are coupling the generation of sequences, which can be derived from next-generation sequencing, to bioinformatics for an optimal selection of sequences for diagnostic antigens. We assumed that although long synthetic peptides are imperfect compared to recombinant proteins of the same length, they are good enough for many purposes. If the coupling efficiency typically is 99% per amino acid, a 100 aa megapeptide is likely to contain at least one error. In a few control experiments we found that megapeptides gave broad peaks in HPLC, indicating inhomogeneity (unpublished data). However, the assumption was that the majority of the molecules would contain enough of correct sequence to be able to mimic also conformational epitopes. The results presented in this paper indicate that this assumption was correct.

Currently, it takes around 70 h to generate an assembled sequence from a sample using Illumina technology. Then, it takes at least 5 h to identify any microbial sequence from this run; in total, around four days. Megapeptides likely to be useful as antigens are then selected and ordered. Peptide synthesis of a 200 aa takes 300 h (13 days). They must then be transported and coupled to SMIA beads (one day). After 18 days, i.e., less than three weeks, a multiplex serology with a reasonable chance of detecting antibodies to the emerging microbe could be in place. Here, we give examples of the utility of megapeptides in suspension multiplex immunoassay systems we currently work with. These are from Filoviruses (Ebola), Herpesvirus 7, and Polyomavirus JC.

Before the antigens can be created, diagnostically-useful peptides must be selected. Unless the location of highly antigenic epitopes is known or highly likely from analogy with related microbes, it is recommendable to synthesize several, e.g., five different megapeptides. The selection of peptide sequences can be from highly-antigenic surface antigens, predicted epitopes, see e.g., the Immune Epitope Database [13], or by knowledge of antigenic proteins from related microbes. Our experience, much of which is unpublished, is that (like for any antigen) megapeptides can work more or less well. Limitations seem often to be due to absence of posttranslational modifications, like glycosylation. However, in this paper we give an example of a megapeptide covering a part of the Ebola surface glycoprotein, which retained specific antigenic activity, albeit lower than a longer glycosylated counterpart. Non-glycosylated proteins, like nucleoproteins or capsid proteins, are sometimes excellently mimicked by megapeptides, e.g., for hantavirus antibody detection (manuscript submitted). By using peptides of over 100 amino acids the likelihood that conformational epitopes can be mimicked is increased. It is our experience that shorter peptides (like 30 aa) are less useful for diagnostic serology (illustrated in Table S2), emphasizing the need for conformational epitopes for this purpose.

The megapeptides tested here were smaller than the complete protein counterparts. The Zaire Ebola glycoprotein (accession nr AHX24667) is 676 amino acids long. HHV7 gB (accession number YP_073779) is 822 amino acids long. JCV VP1 (accession number NP_043511.1) is 354 amino acids long. It is natural that they cannot mimic the antigenicity of the whole molecule. Long recombinant proteins can cover more of the antigenicity. However, megapeptides are simpler to produce and are often cheaper than recombinant proteins on a weight basis. In this paper we could, therefore, use a higher molar concentration of megapeptide than of recombinant protein. From numerous titration experiments with other peptides and megapeptides (unpublished data) we assume that the given conditions for megapeptides are within 50%–100% of the saturation limit of the beads. Like in any immunoassay, the final antigen concentration to be used in an assay ready for routine use must be found by checkerboard titration against suitable antisera. This was not done for this work, whose purpose was proof of principle.

SMIA has several inherent advantages as a serological diagnostic and surveillance system. First, it can combine the antigenicities of several antigens for each targeted microbe. This enables automatic differentiation of true from false positive results, like for a Western or line blot, in a simple test suited for primary testing. The use of several antigens for a single microbe can also avoid false negative results due to target antigen variation, unequal timing of immune responses to different antigens during an infection, or genetic differences in immune response to individual antigens. It is also suitable for syndromic or microbial family-based serological panels. The megapeptide is one of several antigen types which can contribute to these applications.

### 4.2. Megapeptides for Detection of Ebola, HHV7, and JCV Antibodies

In this report, we show results with megapeptides of lengths between 102 and 142 amino acids from viruses of three expanding virus families. In the case of JC polyoma virus VP1 and Human Herpesvirus 7 gB, overlapping megapeptides were used. In the case of Ebola virus, much of the extracellular portion of the surface glycoprotein (the portion most exposed to the immune system) was synthesized. It is likely that, in the future, with further refinement, megapeptides can contribute to improved and quickly-implemented serological diagnosis of these, and other, viruses.

## 5. Conclusions

We conclude that long synthetic peptides can be useful as antigens in microarrays for antibody detection. They can be created relatively simply and quickly just from sequence information.

## Supplementary Materials

The supplementary materials are available online at http://www.mdpi.com/2076-3905/5/3/22/s1.
