# Supplementary material for: Serology in the Digital Age: Using Long Synthetic Peptides Created from Nucleic Acid Sequences as Antigens in Microarrays
Source: Microarrays (Basel). 2016 Aug 10;5(3):22. doi: 10.3390/microarrays5030022 (PMC5040969; doi:10.3390/microarrays5030022)
Supplement: Supplementary file 1 [file microarrays-05-00022-s001.zip › microarrays-135240-SI-check.pdf]

Supplementary Materials: Serology in the Digital Age: Using Long Synthetic Peptides Created from Nucleic Acid Sequences as Antigens in Microarrays

Muhammad Rizwan, Bengt Rönnberg, Maxims Cistjakovs, Åke Lundkvist, Rudiger Pipkorn and Jonas Blomberg

Table S1. Sequences of the megapeptides used in this paper. GenBank identity numbers of the viral proteins, and the position inside the sequence are included for all of them. The megapeptide code name shown in Figures 3 and 4 is shown in parenthesis.

GenBank Identity Number	Position and Name	Sequences
Zaire_Ebola_AHX24667_GP1_385-501	membrane proximal portion of the extracellular part	DNSTHNTPVYKLDISEATQVGQHHRRADNDSTASDTPPATTAAGPLKAENTNTSKSADSLDLATTTSPQNYSETAGNNNTHHQDTGEESASSGKLGLITNTIAGVAGLITGGRRTRR
HHV7_YP_073779_gB_161-260	hhv7gb161	NFKSDTVRRYITTKEPFLRNGPLWFYSTSTSINCIVTDCIAKTKYPFDFFALSTGETVEGSPFYNGINSKTFNEPTEKILFRNNYTMLKTFDDGSKGNFV
HHV7_YP_073779_gB_241-340	hhv7gb241	FRNNYTMLKTFDDGSKGNFVTLTKMAFLEKGNTIFSWEVQNEESSICLLKHWMTIPHALRAENANSFHFIAQELTASFVTGKSNYTLSDSKYNCINSNYT
HHV7_YP_073779_gB_321-420	hhv7gb321	GKSNYTLSDSKYNCINSNYTSILDEIYQTQYNNSHDKNGSYEIFKTEGDLILIWQPLIQRKLTVLENFSNASRKRRKRELETNKDIVYVQLQYLYDTLKD
HHV7_YP_073779_gB_401-500	hhv7gb401	ETNKDIVYVQLQYLYDTLKDYINTALGKLAEAWCLNQKRTITVLHELSKISPSGIISAVYGKPMSAKLIGDVLAVSKCIEVNQTSVQLHKSMRLTKDSSY
HHV7_YP_073779_gB_481-580	hhv7gb481	VNQTSVQLHKSMRLTKDSSYDALRCYSRPLLTYSFANSSKETYLGQLGLDNEILLGNHRTEECEQSNTKIFLSGKFAHIFKDYTYVNSSLITEIEALDAF
HHV7_YP_073779_gB_561-660	hhv7gb561	KDYTYVNSSLITEIEALDAFVDLNIDPLENADFTLLELYTKDELSKANVFDLETILREYNSYKSALHHIETKIATVTPTYIGGIDTFFKGLGALGLGLGA
HHV7_YP_073779_gB_641-740	hhv7gb641	IGGIDTFFKGLGALGLGLGAVLGVTAGALGDVVNGVFSFLKNPFGGALTILLTLGVIGLVIFLFLRHKRLAQTPIDILFPYTSKSTNSVLQATQSVQAQV
HHV7_YP_073779_gB_721-823	hhv7gb721	YTSKSTNSVLQATQSVQAQVKEPLDSSPPYLKTNKDTEPQGDDITHTNEYSQVEALKMLKAIKLLDESYKKAEIAEAKKSQRPSLLERIQYRGYQKLSTEEL

Table S1. *Cont.*

GenBank Identity Number	Position and Name	Sequences
JCV_VP1_1489518_1-102	jcvvp1_1	MAPTKRKGERKDPVQVPKLLIRGGVEVLEVK TGVDSITEVECFLTPEMGDPDEHLRGFSKSISI SDTFESDSPNRDMLPCYSVARIPLPNLNEDLT CGNILM
JCV_VP1_1489518_47-148	jcvvp1_47	EMGDPDEHLRGFSKSISISDTFESDSPNRDML PCYSVARIPLPNLNEDLTCGNILMWEAVTLK TEVIGVTSLMNVHSNGQATHDNGAGKPVQ GTSFHFFSVG
JCV_VP1_1489518_103-218	jcvvp1_103	WEAVTLKTEVIGVTSLMNVHSNGQATHDN GAGKPVQGTSFHFFSVGGEALELQGVLFNYR TKYPDGTIFPKNATVQSQVMNTEHKAYLDK NKAYPVECWVPDPTRNENTRYFGTLT
JCV_VP1_1489518_149-262	jcvvp1_149	GEALELQGVLFNYRTKYPDGTIFPKNATVQS QVMNTEHKAYLDKNKAYPVECWVPDPTRN ENTRYFGTLTGGENVPPVLHITNTATTVLLDE FGVGPLCKGDNLYLSAVDVCGM
JCV_VP1__1489518_219-334	jcvvp1_219	GGENVPPVLHITNTATTVLLDEFGVGPLCKG DNLYLSAVDVCGMFTNRSGSQQWRGLSRYF KVQLRKRRVKNPYPISFLLTDLINRRTPRVDG QPMYGMDAQVEEVRVFEGTEELP
JCV_VP1_1489518_252-354	jcvvp1_252	LYLSAVDVCGMFTNRSGSQQWRGLSRYFKV QLRKRRVKNPYPISFLLTDLINRRTPRVDGQP MYGMDAQVEEVRVFEGTEELPGDPDMMRY VDKYGQLQTKML
